# Machine learning identification of specific changes in myeloid cell phenotype during bloodstream infections

**DOI:** 10.1038/s41598-021-99628-8

**Published:** 2021-10-13

**Authors:** Christian Gosset, Jacques Foguenne, Mickaël Simul, Olivier Tomsin, Hayet Ammar, Nathalie Layios, Paul B. Massion, Pierre Damas, André Gothot

**Affiliations:** 1 University of Liège, Liège, Belgium; 2 Department of Hematobiology and Immuno-Hematology, Liège University Hospital, Liège, Belgium; 3 Intensive Care Unit, Liège University Hospital, Liège, Belgium

**Keywords:** Predictive markers, Predictive medicine, Inflammation, Sepsis, Infection, Dendritic cells, Granulocytes, Monocytes and macrophages, Machine learning, Flow cytometry

## Abstract

The early identification of bacteremia is critical for ensuring appropriate treatment of nosocomial infections in intensive care unit (ICU) patients. The aim of this study was to use flow cytometric data of myeloid cells as a biomarker of bloodstream infection (BSI). An eight-color antibody panel was used to identify seven monocyte and two dendritic cell subsets. In the learning cohort, immunophenotyping was applied to (1) control subjects, (2) postoperative heart surgery patients, as a model of noninfectious inflammatory responses, and (3) blood culture-positive patients. Of the complex changes in the myeloid cell phenotype, a decrease in myeloid and plasmacytoid dendritic cell numbers, increase in CD14^+^CD16^+^ inflammatory monocyte numbers, and upregulation of neutrophils CD64 and CD123 expression were prominent in BSI patients. An extreme gradient boosting (XGBoost) algorithm called the “infection detection and ranging score” (iDAR), ranging from 0 to 100, was developed to identify infection-specific changes in 101 phenotypic variables related to neutrophils, monocytes and dendritic cells. The tenfold cross-validation achieved an area under the receiver operating characteristic (AUROC) of 0.988 (95% CI 0.985–1) for the detection of bacteremic patients. In an out-of-sample, in-house validation, iDAR achieved an AUROC of 0.85 (95% CI 0.71–0.98) in differentiating localized from bloodstream infection and 0.95 (95% CI 0.89–1) in discriminating infected from noninfected ICU patients. In conclusion, a machine learning approach was used to translate the changes in myeloid cell phenotype in response to infection into a score that could identify bacteremia with high specificity in ICU patients.

## Introduction

Sepsis is a leading cause of morbidity and mortality and causes a considerable economic burden^1^. Sepsis involves complex biological processes caused by the colonization of sterile tissue or fluid by microorganisms, leading to multiple organ failure and death^2,3^. Each year, the number of sepsis cases and sepsis-related deaths increases dramatically^4,5^. Early support and management of sepsis is thus a major public health issue^6^. Treatment of infections is often delayed because of the overall 24–48 h turn-around time required for a blood culture to become positive^7^. In addition, almost half of blood cultures from patients with clinically defined sepsis remain negative^8,9^, which may lead to initial undertreatment of suspected infections^4^.

Predicting and monitoring infection severity require accurate and specific biomarkers. Commonly used biomarkers include C-reactive protein (CRP) and procalcitonin (PCT)^10,11^. The predictive values of CRP and PCT reported in the literature can vary substantially. These biomarkers are not pathognomonic of infection, as their levels are also increased in noninfectious inflammatory states^12–15^. Additionally, the elevation of CRP/PCT levels may vary with patient liver function. However, in the context of a proven infection, CRP/PCT kinetics may be used for patient management.

Developing risk prediction scores has been an area of significant interest, as they can be used to support medical decisions for infected patients^16–21^. Machine learning (ML) algorithms, which belong to the field of artificial intelligence, have been devised using arrays of clinical and/or biological data to increase diagnostic and prognostic accuracy^22^.

Studies have been conducted to improve bloodstream infection (BSI) prediction models by including novel risk markers^23^. Better model performance can be achieved by ensemble methods that use multiple learning algorithms as opposed to the performance achieved by any single model taken separately^24–28^.

The need to process vast amounts of information and make them exploitable in the clinic remains a current challenge. Logistic regression can be used for simple clinical applications, but it may not be precise enough for use in the intensive care unit because individual responses to disease generate nonlinear relationships and complex model interactions^29^. Gradient boosting technology is therefore well suited to identify chaotic behavior among predictors^30^. The extreme gradient boosting methodology^31^ (XGBoost), which is used for classification and regression, has recently become one of the most favored learning machines and is widely used by data scientists at all levels of expertise^32,33^, particularly in sepsis prediction^34–36^.

Here, we designed a method to predict and monitor the development of sepsis by measuring the expression of cell surface markers on myeloid cell populations, including neutrophils, monocytes and dendritic cells. The selection of biomarkers was based on a literature survey of monocyte markers whose expression was reported to be influenced by sepsis and/or infection. Comprehensive monocyte phenotyping was performed in preliminary studies (data not shown). A final marker panel was selected such that it could be implemented in a single 8-color protocol.

An ML approach was used to design a score intended to identify bloodstream infections in the ICU and differentiate noninfectious from infectious inflammatory states. The prognostic value of the phenotypic score was also assessed. Immunophenotypes were measured in healthy individuals, heart surgery patients (as a model of noninfectious inflammatory state) and patients with positive blood cultures at different time points.

## Methods

### Patient selection and data collection

All the methods were performed in accordance with the relevant guidelines and regulations established by the institutional review board. Previously collected blood samples of patients were acquired following a protocol approved by the “Comité d’Ethique médicale hospitalo-facultaire universitaire de Liège (707)” in Liège, Belgium (approval # 2018/309). The patients or their relatives were informed that previously collected body materials sampled for routine care may be used for research laboratory tests in an anonymous manner unless they expressed their opposition. Informed consent was obtained from all the participants or their relatives. In addition, elective blood samples were drawn from 46 asymptomatic healthy individuals from among institute staff and students, from which written informed consent was obtained. These individuals had no medical complaints at the time of analysis and thus were assumed to be BSI-free and used as controls.

The patient population consisted of two groups. The first group comprised heart surgery patients sampled at 2, 24 and 48 h after surgery and treated with preventive cefuroxime-based antibiotic therapy for the first 24 h. Only patients without any suspicion or evidence of infection were included. At 48 h, only those with a CRP level higher than 180 mg/l were included in the study to maximize the phenotypic changes during a severe nonseptic inflammatory response. The second group included 60 BSI patients whose immunophenotype was scored up to 8 h after the microbiological blood culture became positive.

In a further validation study, blood samples from 62 ICU patients were collected as per the physician’s request and scored by the laboratory in a blinded fashion. Validation cases were categorized as infectious cases when the presence of a pathogen was documented using microbiological cultures and the patient was treated with antibiotic therapy. Electronic medical record (EMR) data, i.e., the sequential organ failure assessment (SOFA) score and simplified acute physiology score (SAPS) II, were calculated and recorded by a data manager and communicated to the research team.

### Microbiological blood cultures

Blood cultures were set up in a BacT/Alert system (Biomérieux, Marcy l’Etoile, France), which is based on the colorimetric detection of CO_2_ released by the growth of microorganisms. Germ identification was performed by matrix-assisted laser desorption ionization time-of-flight (MALDI-TOF) analysis (Biotyper, Brüker, Billerica, MA, USA). In some cases, further identification tests were carried out on a Vitek-2 system (Biomérieux), which was also used for antibiogram acquisition.

Blood samples were drawn in pairs from the venous peripheral line and the arterial line. Only patients with positive and concordant results were considered as having BSI.

### Blood sample processing and data acquisition by flow cytometry

An 8-color monoclonal antibody cocktail consisting of anti-CD14 (CD14 APC-H7, BD Biosciences, San Jose, CA, USA, clone MρP9, 5 µl), anti-CD16 (CD16 PE BD Biosciences, clone 3G8, 20 µl), anti-CD45 (CD45 V500, BD Bioscience, clone HI30, 5 µl), anti-CD64 (CD64 PE-Cy7, BD Biosciences; clone 10.1, 5 µl), anti-CD91 (CD91 PerCP-efluor710, ThermoFisher, Waltham, MA, USA, clone A2MR-a2, 5 µl), anti-CD123 (CD123 APC, Sony Biotechnology, San Jose, CA, USA, clone 6H6, 5 µl), anti-HLA-DR (HLA-DR FITC, Sony Biotechnology, clone L243, 5 µl) and anti-Integrin β7 (Integrin β7 BV421, BD Biosciences, clone FIB504, 2.5 µl) was prepared in 12 × 75 mm polystyrene tubes (BD Biosciences). A volume of 100 µl EDTA-treated whole blood was added, gently mixed and incubated for 20 min in the dark at room temperature. Then, red blood cells were lysed by adding 2.5 ml of BD FACS lysing solution per sample and incubated for 12 min in the dark at room temperature. The cells were then washed twice by centrifugation at 700 × g for 5 min, resuspended in 500 µl BD cell wash medium and stored at 4 °C until analysis. To prevent the degradation of the phenotypic markers, all the samples were processed within 6 h of collection.

In parallel, total leukocytes were measured with a Sysmex XS-800 hematology analyzer (Kobe, Japan) for quantification of absolute cell counts.

**Figure 1:**
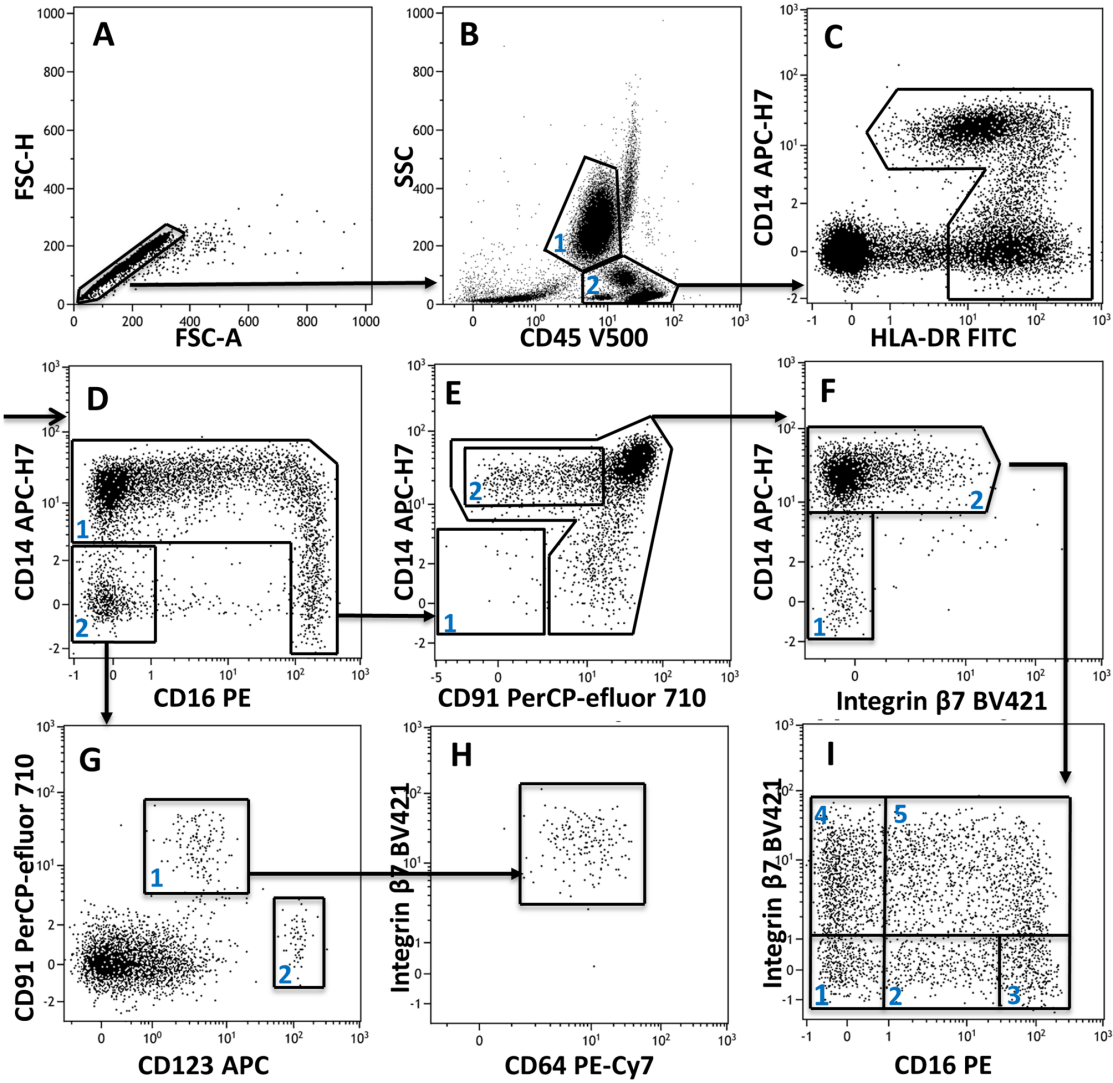
Sequential gating was used to isolate 7 distinct monocyte and 2 dendritic cell subsets (explanations in the text). The example shown is from a cardiac surgery patient 24 h postoperatively.

Immunophenotypic data were acquired using a FACS Canto II (BD Biosciences, San Jose, CA, USA) flow cytometer with BD FACS DIVA software v8.0.1. Eight-peak calibration particles (Rainbow Beads, BD) were used to align PMT voltages for measurements and flow cytometer setup alignments. On a daily basis, Cyto-Cal™ calibration beads were used to monitor the stability and sensitivity of the flow cytometer, while BD cytometer setup and tracking (CS&T) beads were used to set the PMT gain for all fluorescence channels ensuring day-to-day stability of median fluorescence intensities. Spectral spillover of fluorochrome signals was corrected through a compensation matrix generated by single-stained compensation beads (FacsComp, BD Biosciences). Data acquisition was performed within 1 h of staining. Six thousand monocyte events were recorded per sample based on a gate created on bivariate scatterplots of forward vs. side scatter. Flow cytometric data were exported as FCS files and analyzed using Kaluza software (v2.1, Beckman Coulter, Brea CA, USA). The template shown in Fig. 1 was used to delineate monocyte and dendritic cell populations and was manually adjusted for each sample. Mean fluorescence intensities (MFIs) of cell surface markers, percentages (%) of different cell subpopulations and quantitative cell counts (number of cells/µl) were computed. XGBoost construction used a total of 101 variables. MFIs of 8 phenotypic markers were calculated on the following 10 cell populations: mononuclear cells, neutrophils, total monocytes and 7 subclasses of monocytes, which totaled 80 parameters. Cell class percentages were calculated for 9 populations, i.e., pDCs, mDCs and the 7 classes of monocytes. Cell counts of pDCs, mDCs, total monocytes and 7 monocyte subpopulations were also included in the model (for a complete list of the variables, see Appendix 1). After extracting the myeloid cell population data in Kaluza, the data were exported as a .csv file.

### Identification of monocyte and dendritic cell subsets

A set of surface markers expressed in myeloid cells was selected because their expression was expected to be modulated during infectious processes. The following biomarkers were measured: human leukocyte antigen-DR (HLA-DR), integrin β7 subunit, TLR-4 coreceptor (CD14), type III Fcγ receptor (CD16), leukocyte common antigen (CD45), type I Fcγ receptor (CD64), low-density lipoprotein receptor-related protein 1 or LRP1 (CD91), and IL-3 receptor α chain (CD123). Singlets were first selected using FSC-A/FSC-H dot plot (Fig. 1A). Neutrophils were gated away from monocytes based on their high side scatter (SSC) and lower surface expression of CD45 (Fig. 1B gate 1). Next, we created a gate of mononuclear cells (Fig. 1B gate 2) that was further analyzed with a combination of CD14 and HLA-DR to select HLA-DR-expressing cells (Fig. 1C). An overall monocyte gate was constructed around classical CD14^+^CD16^-^ monocytes, intermediate CD14^+^ CD16^+^ monocytes and nonclassical CD14^low^ CD16^+^ monocytes (Fig. 1D gate 1), while dendritic cells, HLA-DR-positive CD16^-^ NK cells, B cells, and HLA-DR-positive T lymphocytes were gated out (Fig. 1D gate 2). The remaining lymphoid cells were eliminated as CD14^-^ CD91^-^ cells (Fig. 1E gate 1). The monocyte population was further subdivided by first isolating a population with low CD91 expression (Fig. 1E gate 2). The resulting CD14 + monocyte population (Fig. 1F gate 2) was subdivided based on integrin β7 and CD16 expression into five distinct CD14^+^ CD91^+^ monocyte populations (Fig. 1I gates 1–5), while a seventh monocyte subset included CD14^low^β7^-^ cells (Fig. 1F gate 1). Myeloid dendritic cells DCs (mDCs) and plasmacytoid dendritic cells (pDCs) were also isolated. Both DCs and monocytes expressed HLA-DR, while dendritic cells did not express CD14 and CD16 (Fig. 1D gate 2). The dendritic cell subpopulations were segregated by differential expression of CD91 and CD123 (Fig. 1G gate 1-mDCs, gate 2-pDCs). The expression of CD64 and integrin β7 was also present on mDCs (Fig. 1H).

### Algorithm training

After classifying the patients into groups, we performed extreme gradient amplification (XGBoost) 32 times on a random sample using bootstrap technology for classification algorithms. Thirty-two XGBoost bootstrap sets were formed on the 101 variables representing antigen MFI, cell population percentages and absolute counts (Appendix 2, A). An optimal parameter search indicated that the best model performance was achieved when all the variables were included in the algorithm design. Cross-validation was used to both train the algorithm with all the data and then indirectly test it with all the data^37^.

The variable importance feature rankings were charted to evaluate how the different variables affect the model predictions (Appendix 1). To evaluate the variability of the performance main model, 30 repeated tenfold cross validation models with different random seeds were reported (Appendix 2, B). In addition, 30 shuffled models were run in a repeated process to obtain good estimates of model performance relative to randomized data (Appendix 2, C). The performance of the bootstrapped ensemble models with parameter optimization compared to our final model is presented in Appendix 3. The main parameters of the distribution of models with mean trend, median, measure of variance, standard deviation, minimum, maximum and standard error are presented in Appendix 3.

### Score construction

A classification system was modeled to evaluate the modulation of biomarkers generated by the infectious state, independent of inflammation. Healthy individuals and heart surgery patients were pooled into a single class and classified as noninfectious, while patients with positive blood cultures were labeled as infectious. Infected and noninfected individuals were categorized according to a binary (0–1) classification system that returned a probability of systemic infection.

### iDAR score

The iDAR score is an acronym for “infection detection and ranging score”. iDAR integrates 32 classification models built from a bootstrap sampling of the data. An ensemble probability of infection was calculated. First, we averaged the responses of the bootstrap models, which yielded the mean response (r) on the half log-odds scale. We applied the formula $p=\frac{{e}^{-2r}}{1+{e}^{-2r}}$ to establish the probability associated with overall mean response. The final result was then multiplied by a factor of 100 to generate an estimate of infection risk, expressed as a percentage (Appendix 4).

### Statistical analysis

Differences between independent groups were considered significant at *p* < 0.05 using Mann–Whitney tests. The relationship between two variables was established using Spearman correlation analysis. The sensitivity and specificity of the models were analyzed using ROC curves for classification purposes. Statistical analysis was carried out using the Salford Predictive Modeler 8.3 (Minitab Ltd., State College, PA, USA) and GraphPad Prism 6 (GraphPad Software, San Diego, CA, USA) software packages.

## Results

### Patient characteristics

The study cohort comprising a total of 251 subjects was divided into three groups. The first group included 46 healthy subjects. The second group included 145 patients who had undergone cardiac surgery, sampled at three different postoperative times (2 h, 24 h, and 48 h with a CRP > 180 mg/l). The third group included 60 patients with positive blood cultures. For each subject, the immunophenotypic profile, C-reactive protein levels and blood cell counts were recorded (Table 1). Mortality in heart surgery patients was 5.3%, with a median hospital stay of 12 days. The majority of cardiac surgery patients were male (72%), with a median age of 69 years, which was higher than the median age of the blood culture patients (66 years). There was also a gender imbalance in favor of males among patients with positive blood cultures (62% vs. 38%). The proportion of BSI patients receiving empirical antibiotic therapy prior to blood collection was 72%. The pathogens most frequently identified in blood cultures were *Escherichia coli* (34%), *Klebsiella pneumoniae* (17%), *Staphylococcus aureus* (15%), *Staphylococcus epidermidis* (8%) and *Streptococcus pneumoniae* (5%), representing 79% of the bacteria identified in blood cultures. Additional pathogens (< 4%) included *Klebsiella oxytoca*, *Pseudomonas aeruginosa*, *Staphylococcus hominis*, *Streptococcus agalactiae*, *Enterococcus faecalis*, *Enterococcus faecium*, *Streptococcus oralis*, *Serratia marcescens*, *Enterobacter cloacae* and *Staphylococcus capitis*. Of the 60 patients with a positive blood culture, 17 (28.3%) patients were reported in EMR records as having sepsis, 8 (13.3%) had septic shock and 6 (10%) had endocarditis. Patients with positive blood cultures had a median hospital stay of 31 days, with a mortality rate of 30.5%. The mortality rate was 17% in patients with sepsis, 50% in those with septic shock and 83% in endocarditis.

**Table 1:** Demographic and laboratory characteristics of patients.

Characteristics	Healthy (n = 46)	Cardiac surgery (n = 145)			Bloodstream infections (n = 60)
		H_2_ (n = 22)	H_24_ (n = 80)	H_48_ (n = 43)	
C-Reactive Protein mg/l (Mean ± SEM)	< 5	3.4 ± 0.9	69 ± 3.8	242 ± 6.5	218 ± 15
Neutrophil count/µl (Mean ± SEM)	3368 ± 113	8687 ± 633	9944 ± 334	8706 ± 387	13,543 ± 891
Lymphocyte count/µl (Mean ± SEM)	2016 ± 72	1792 ± 155	922 ± 48	1296 ± 69	999 ± 84
Monocyte count/µl (Mean ± SEM)	561 ± 23	682 ± 67	1071 ± 46	1173 ± 57	1040 ± 88
Age (median years)	43	69			66
Gender M/F (%)	58/42	72/28			62/38
Hospitalization length (median days)	–	12			31
Decease after admission (median days)	–	–			61
Hospitalized mortality	–	5.3%			30.5%
**Site of infection**					
Catheter					3%
Surgical site					7%
Endocarditis					10%
Abdominal					15%
Lung					18%
Other					22%
Urinary tract					25%
**Cause of hospital admission**					
Carcinoma					3%
General deterioration					3%
Cerebrovascular accident					7%
Digestive tract hemorrhage					7%
Trauma					7%
Hematological malignancy					7%
Respiratory failure					7%
Heart failure					8%
Renal insufficiency					8%
Postsurgery monitoring					10%
Localized infection—Sepsis—Septic Shock					33%

### Monocyte and dendritic cell subset redistribution in inflammation and infection

**Figure 2:**
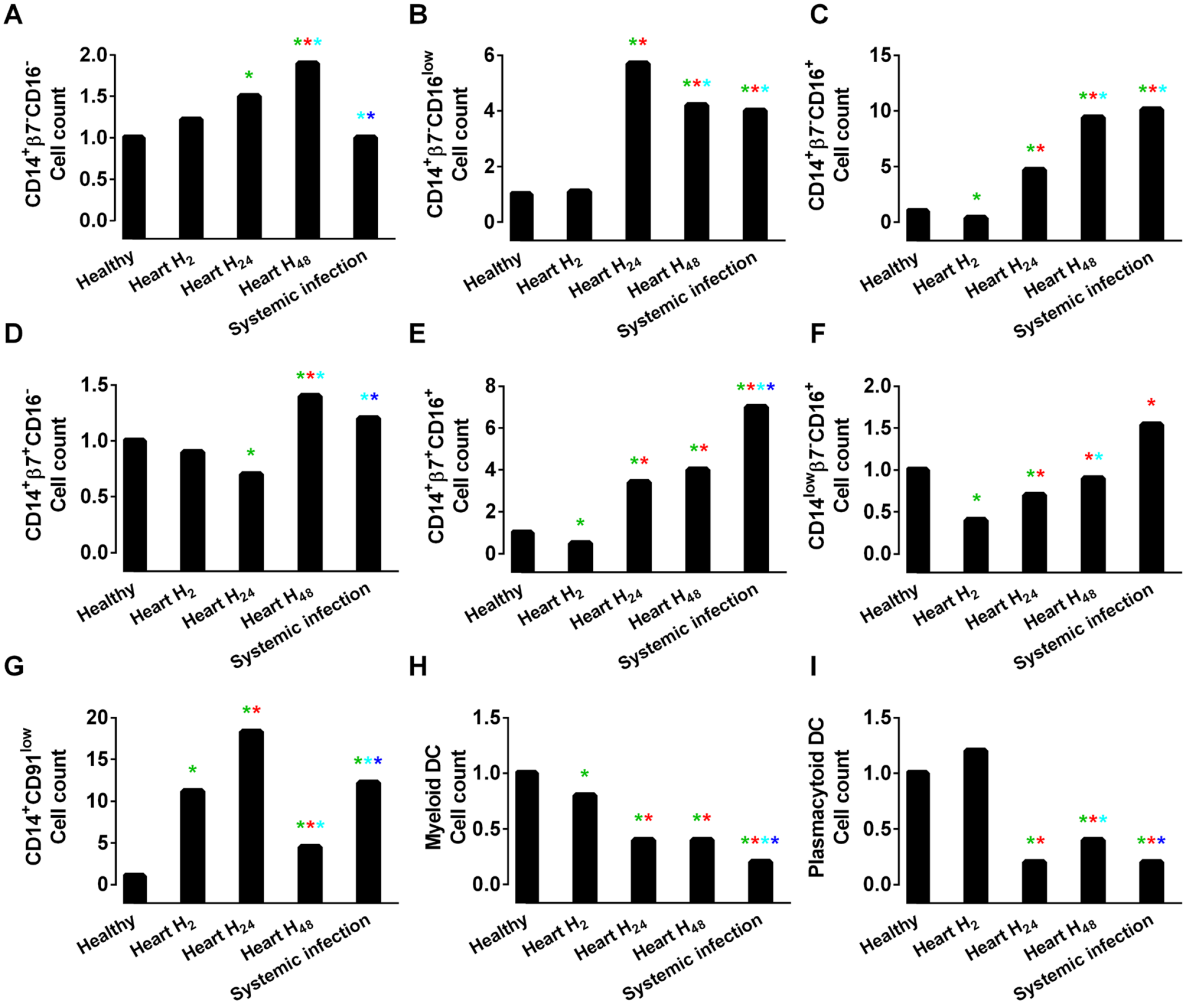
Monocyte and dendritic cell counts for CD14^+^β7^-^CD16^-^ monocytes (**A**), CD14^+^β7^-^CD16^low^ monocytes (**B**), CD14^+^β7^-^CD16^+^ monocytes (**C**), CD14^+^β7^+^CD16^-^ monocytes (**D**), CD14^+^β7^+^CD16^+^ monocytes (**E**), CD14^low^β7^-^CD16^+^ monocytes (**F**), CD14^+^CD91^low^ monocytes (**G**), myeloid dendritic cells (**H**) and plasmacytoid dendritic cells (**I**). The data are presented as the mean ± standard error of the mean (SEM) in healthy, cardiac surgery patients at 2 (H_2_), 24 (H_24_), 48 (H_48_) hours and systemically infected patients. The data scale was normalized to reflect the relative increase or decrease compared to healthy subjects. *P* < 0.05 vs. Healthy “green asterisk” vs. Heart H_2_“red asterisk”; vs. Heart H_24_“light blue asterisk”; vs. Heart H_48_“dark blue asterisk”.

The distribution of monocyte and dendritic cell subsets was evaluated in patients at H_2_, H_24_, and H_48_ after cardiac surgery and in BSI patients compared to healthy individuals. Figure 2 shows the fold-changes in the number of cells per microliter of the monocyte and dendritic cell populations compared to the control group.

The highest variation was observed for CD14^+^CD91^low^ monocyte numbers, which increased up to 20-fold in postsurgery H_24_ patients and remained elevated more than tenfold in BSI over time. The subset of CD14^+^β7^-^CD16^+^ monocytes expanded gradually up to tenfold postsurgery and was even slightly larger in systemic infection patients. The β7^+^ counterpart, i.e., CD14^+^β7^+^CD16^+^ monocyte numbers, was also increased in surgical patients and was maximal in bloodstream infection (sevenfold increase compared to controls). Additionally, the augmentation of CD14^+^β7^-^CD16^low^ monocyte numbers was notable and reached a sixfold change at 24 h postsurgery and a fourfold change in systemic infection patients. For dendritic cell subpopulations, it was striking that the numbers decreased in aseptic and especially in septic inflammation, during which a fourfold nadir was observed for both myeloid and plasmacytoid DCs. Overall, compared to postsurgery patients and controls, systemic infection patients were discriminated by maximal increases in the numbers of CD14^+^β7^-^CD16^+^ monocytes, CD14^+^β7^+^CD16^+^ monocytes, and CD14^low^β7^-^CD16^+^ monocytes and a significant decrease in the numbers of myeloid DCs.

Flow cytometry plots allowing visual representation of myeloid cell subset redistribution during postsurgery inflammation and systemic infection are presented in Appendix 5.

### ML modeling of myeloid cell phenotype changes associated with BSI

Overall, BSI can be differentiated from sterile inflammation by complex shifts in monocyte and DC subpopulations, which we intended to model using an ML algorithm. The objective was to summarize the complexity of immunophenotypic changes into a simple score that would be used to predict the risk of BSI.

**Figure 3:**
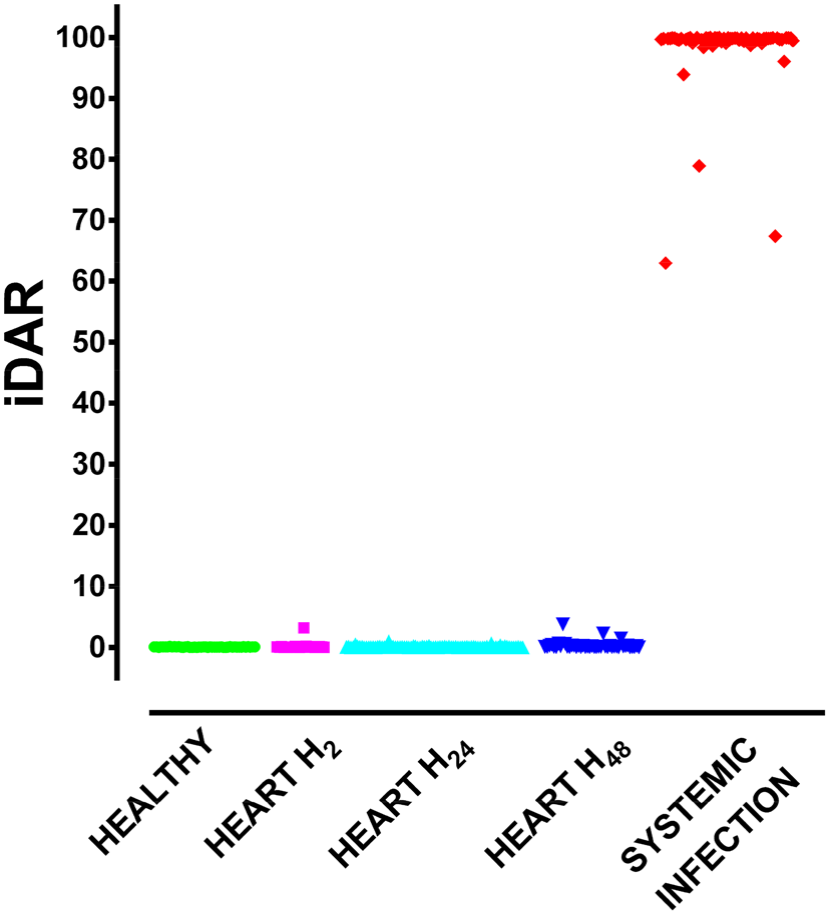
iDAR score values in the 5 patient groups of the discovery cohort.

The dataset was divided into 5 groups: 46 healthy patients, 22 H_2_, 80 H_24_, 43 H_48_ cardiac surgery patients and 59 patients with positive blood cultures. Optimization of the hyperparameters for the classification was initially carried out for model stability. The 32 bootstrap set classification algorithms with tenfold cross-validation showed an average AUROC of 0.988 for the detection of blood culture-positive patients with a specificity of 98% and a sensitivity of 92.1% at a probability cutoff value of 24%. When considering repeated observations of subjects, it is desirable to assign subjects rather than individual data records to cross validation bins, clustering all the data belonging to a given subject at all times, which in our case is based on a temporal analysis of patient blood samples. In the presence of data with a temporal dimension, it may be useful to distribute the data in bins along the temporal dimension in search of possible bias in the blood sampling methodology. In this case, we found that the two cross-validation bootstrap models for the bootstrap classification models maintained similar performance (AUC classification: 0.983 vs. 0.988), which ensured that the sampling criteria were not disrupted over time. A randomization test was also performed, and the area under the receiver operating curve (AUROC = 0.58) in that configuration showed the validity of our bootstrapped models with respect to the null hypothesis. Additional statistical information for each algorithm and set of characteristics related to the importance of variables and the accuracy of XGBoost modeling are found in Appendices 1–3. The algorithm was scored for each individual (Fig. 3), which provides evidence that the iDAR score provides a clear segregation of infectious versus noninfectious inflammatory states.

### Identification of the most important predictors of the iDAR score

The iDAR score represents variations in 101 variables in BSI. To assess their relative importance, the 10 most important predictors (Appendix 3**)** were identified using the relative variable importance score (RVIS), which was measured by 10 randomization permutation tests. The RVIS rescales the importance of variables on a 100-point scale (the most important variable always obtains a value of 100). Any additional variable is rescaled to reflect its importance in relation to the most important variable. Variable significance analysis suggests that the MFI of CD14 on CD14^+^β7^-^CD16^+^ monocytes (RVIS = 100), MFI of CD64 on neutrophils (RVIS = 75.5), MFI of CD64 on CD14^-^β7^-^CD16^+^ monocytes (RVIS = 51), MFI of integrin β7 on CD14^+^CD91^low^ monocytes (RVIS = 6.8), percentage and absolute count of myeloid dendritic cells (RVIS = 12; 3.9), MFI of CD14 on CD14^low^ β7^-^CD16^+^ (RVIS = 3.1), percentage and MFI HLA-DR on CD14^+^CD91^low^ cells (RVIS = 2.2; 2.1) and the expression of CD16 on monocytes (RVIS = 2) were the most valuable markers for identifying ‘BSI present”.

The partial dependence diagrams show a positive correlation of infection with the following variables: MFI of CD64 on CD14^low^β7^-^CD16^+^ monocytes and neutrophils, MFI of CD14 on CD14^low^β7^-^CD16^+^ monocytes, MFI of CD16 on monocytes, percentage and MFI of integrin β7 on CD14^+^CD91^low^ monocytes. An inverse correlation with infection was observed for the percentage and absolute counts of myeloid dendritic cells and plasmacytoid dendritic cells, MFI of CD14 on CD14^+^β7^-^CD16^+^ monocytes and expression of HLA-DR on CD14^+^CD91^low^ monocytes (Appendix 2D).

### Variations in myeloid antigen densities during BSI and postsurgery inflammation

**Figure 4:**
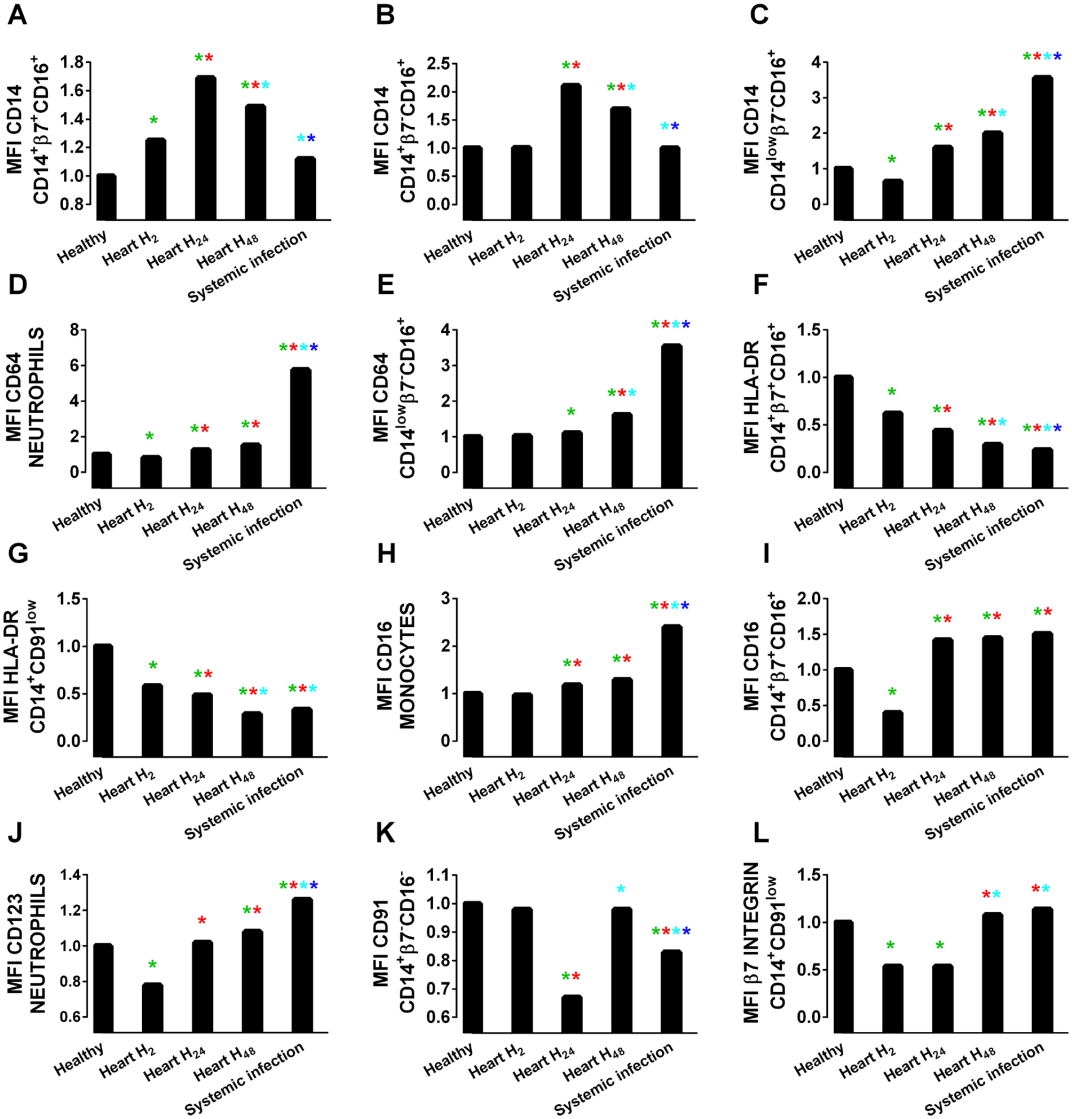
The 12 most relevant MFIs of selected markers for iDAR score construction. (**A**), CD14 expression on CD14^+^β7^+^CD16^+^ monocytes (**B**), CD14 expression on CD14^+^β7^-^CD16^+^ monocytes (**C**), CD14 expression on CD14^low^β7^-^CD16^+^ monocytes (**D**), CD64 expression on neutrophils (**E**), CD64 expression on CD14^low^β7^-^CD16^+^ monocytes (**F**), HLA-DR expression on CD14^+^β7^+^CD16^+^ monocytes (**G**), HLA-DR expression on CD14^+^CD91^low^ monocytes (**H**), CD16 expression on monocytes (**I**), CD16 expression on CD14^+^β7^+^CD16^+^ monocytes (**J**), CD123 expression on neutrophils (**K**), CD91 expression on CD14^+^β7^-^CD16^-^ monocytes (**L**), and integrin β7 expression on CD14^+^CD91^low^ monocytes in the indicated patient groups versus healthy donors. The data are presented as the mean ± standard error of the mean (SEM) in healthy, cardiac surgery patients at 2 (H_2_), 24 (H_24_), 48 (H_48_) hours and BSI patients. The data scale was normalized to reflect the relative increase or decrease compared to healthy persons. *P* < 0.05 Healthy “green asterisk”; Heart H_2_“red asterisk”; Heart H_24_“light blue asterisk”; Heart H_48_“dark blue asterisk”.

As seen in Appendix 3, many of the most important predictors in the iDAR score are expression intensities—expressed as MFI—of various antigens in specific cell populations. A comparison of the 12 most relevant MFIs was carried out between patient groups and healthy donors (Fig. 4). The CD14 MFI was up to twofold higher for both the β7^+^ (Fig. 4A) and β7^-^ (Fig. 4B) CD14^+^CD16^+^ monocyte subsets at 24 h and 48 h postsurgery than that of the controls but was unchanged in BSI patients. In contrast, in CD14^low^β7^-^CD16^+^ monocytes, CD14 MFI increased in both postoperative patients (twofold) and BSI patients (fourfold) (Fig. 4C). Neutrophils (Fig. 4D) and CD14^low^β7^-^CD16^+^ monocytes (Fig. 4E) overexpressed CD64 in BSI patients by threefold and sixfold, respectively, while much smaller variations were seen in postoperative patients. The expression of HLA-DR declined in CD14^+^β7^+^CD16^+^ (Fig. 4F) and CD14^+^CD91^low^ (Fig. 4G) monocytes over the postoperative period from H_2_ to H_48_ and was also depressed in BSI patients. CD16 expression on total monocytes (Fig. 4H) was slightly elevated in postoperative patients and more significantly elevated in those with BSI. On CD14^+^β7^+^CD16^+^ monocytes (Fig. 4I), CD16 was initially depressed at H_2_ postoperatively and then increased at H_24_ and H_48_, as well as in BSI. A similar pattern was observed for the expression of CD123 on neutrophils (Fig. 4J). CD91 expression on CD14^+^β7^-^CD16^-^ decreased in cardiac surgery patients after 24 h and then returned to normal levels at 48 h; CD91 expression in that cell population was also decreased in BSI patients (Fig. 4K). Integrin β7 expression was initially decreased at 2 and 24 h after cardiac surgery and returned to control levels at 48 h but was not significantly modified during BSI (Fig. 4L).

### In-house, out-of-sample validation of the iDAR phenotypic score

In the final step of the present study, we carried out an evaluation of the iDAR score in a different cohort of ICU patients. The score was evaluated in sixty-two ICU patients referred to the laboratory in a blinded fashion and subsequently subdivided into 3 groups based on the absence of documented infection (Group 1, 29 patients), localized infections without positive blood culture (Group 2, 16 patients) and patients with positive blood culture (Group 3, 17 patients). The iDAR, SOFA and SAPS II scores, together with CRP level (mg/l), temperature (°C) CD64 MFI on neutrophils and lactate levels (mg/l), were determined.

**Figure 5:**
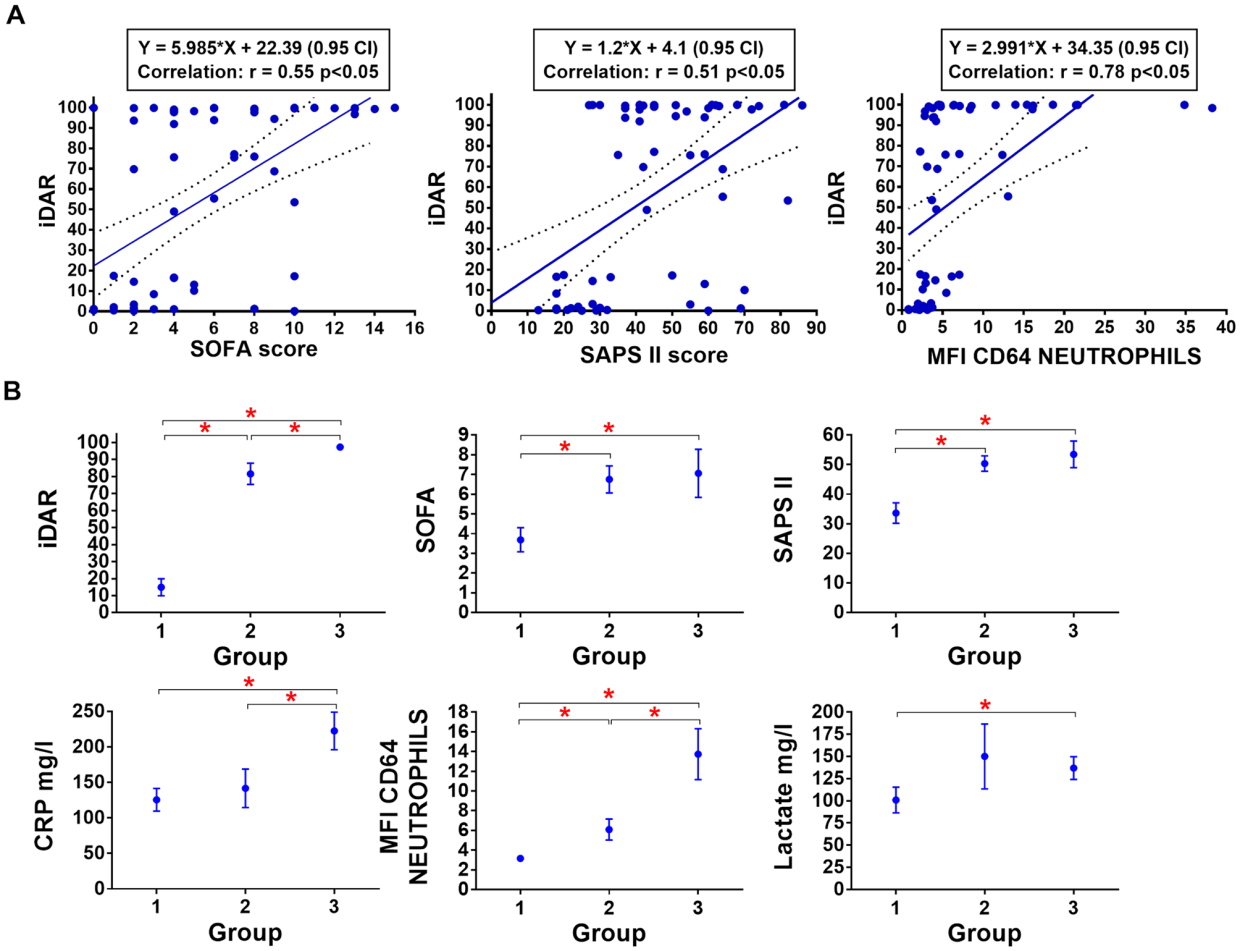
(**A**) Correlation between iDAR and SOFA, SAPS II or CD64 MFI (**B**). iDAR, SOFA, SAPS II, CRP, CD64 MFI and lactate levels in the validation cohort (mean ± SEM): Group 1, patients with no documented infection; Group 2, patients with a documented localized infection; Group 3, patients with systemic infection. *p < 0.05.

A close correlation was found between the SOFA score and the SAPS II score (corr = 0.73), as they share common characteristics (Fig. 5A). Compared to iDAR, the highest correlation was found with CD64 MFI on neutrophils (corr = 0.78), followed by the SOFA score (corr = 0.55), SAPS II score (corr = 0.51), CRP levels (corr = 0.32) and lactate levels (corr = 0.32), *p* < 0.05. No significant correlation was found between iDAR and temperature (corr = − 0.04) or mortality.

Overall, iDAR and CRP were higher in BSI patients than in localized infectious and noninfectious patients (*p* < 0.05). In addition, iDAR was significantly increased in patients with systemic infections compared to localized infections (*p* < 0.05), while the CRP levels were not different (*p* > 0.05). Infected patients had higher SOFA and SAPS II scores than noninfected patients (*p* < 0.05), but there was no difference between patients with localized and systemic infections. Lactate levels were increased in BSI patients (*p* < 0.05) compared to noninfected patients but did not discriminate localized from systemic infections (Fig. 5B). Patient temperature measurements did not vary between groups.

AUROC, specificity, sensitivity and cutoff values were calculated first for the discrimination of noninfected patients and infected patients, i.e., Group 1 versus Groups 2 and 3. In that configuration, the performance of the iDAR score was excellent, with an AUROC of 0.95 and a sensitivity/specificity higher than 90% at a threshold of 54.5. The performance of the score was also assessed for the discrimination of localized and systemic infection, i.e., Group 2 versus 3. Again, with an AUROC of 0.85, the iDAR score could accurately classify these two categories with a sensitivity/specificity higher than 80% at a cutoff of 98.4. On the other hand, the AUROCs of SAPS II, lactate, CRP, SOFA, and temperature were below 0.8 (data not shown). In addition, we confirmed the performance of CD64 MFI on neutrophils. AUROC was 0.84 for the detection of infection (Group 1 versus Groups 2 and 3) and 0.76 for localized and systemic infection discrimination (Group 2 versus Group 3). Sensitivity and specificity were inferior to those achieved with iDAR (Table 2).

**Table 2:** Validation in iDAR in 3 ICU subgroups.

Model validation	AUROC	Sensitivity	Specificity	PPV	NPV	Cutoff
**iDAR**						
Group 1 vs. 2 + 3	0.95 (95% CI 0.89–1)	97%	93%	94%	96%	> 54.5
Group 2 vs. 3	0.85 (95% CI 0.71–0.98)	82%	81%	82%	81%	> 98.4
**MFI CD64 Neutrophils**						
Group 1 vs. 2 + 3	0.84 (95% CI 0.75–0.94)	70%	83%	82%	71%	> 4.3
Group 2 vs. 3	0.76 (95% CI 0.59–0.92)	59%	81%	77%	65%	> 8.5

AUROC, sensitivity, specificity, positive and negative predictive values (PPV, NPV) are shown at the indicated cutoffs. The performance of CD64 MFI on the same cohort is presented for comparison.

The model was also evaluated after elimination of coagulase-negative staphylococci, i.e., *Staphylococcus epidermidis*, *Staphylococcus hominis* and *Staphylococcus capitis,* which are usually considered contaminants. These species accounted for 11% of the bacteria found in our blood cultures. AUROC values remained unchanged when comparing Group 1 with Groups 2 and 3 or Group 2 with Group 3. Indeed, 10% influence trimming was included in our model building, which eliminates training data that are far from the decision threshold.

### Examples of monitoring patients in the ICU according to the iDAR score

**Figure 6:**
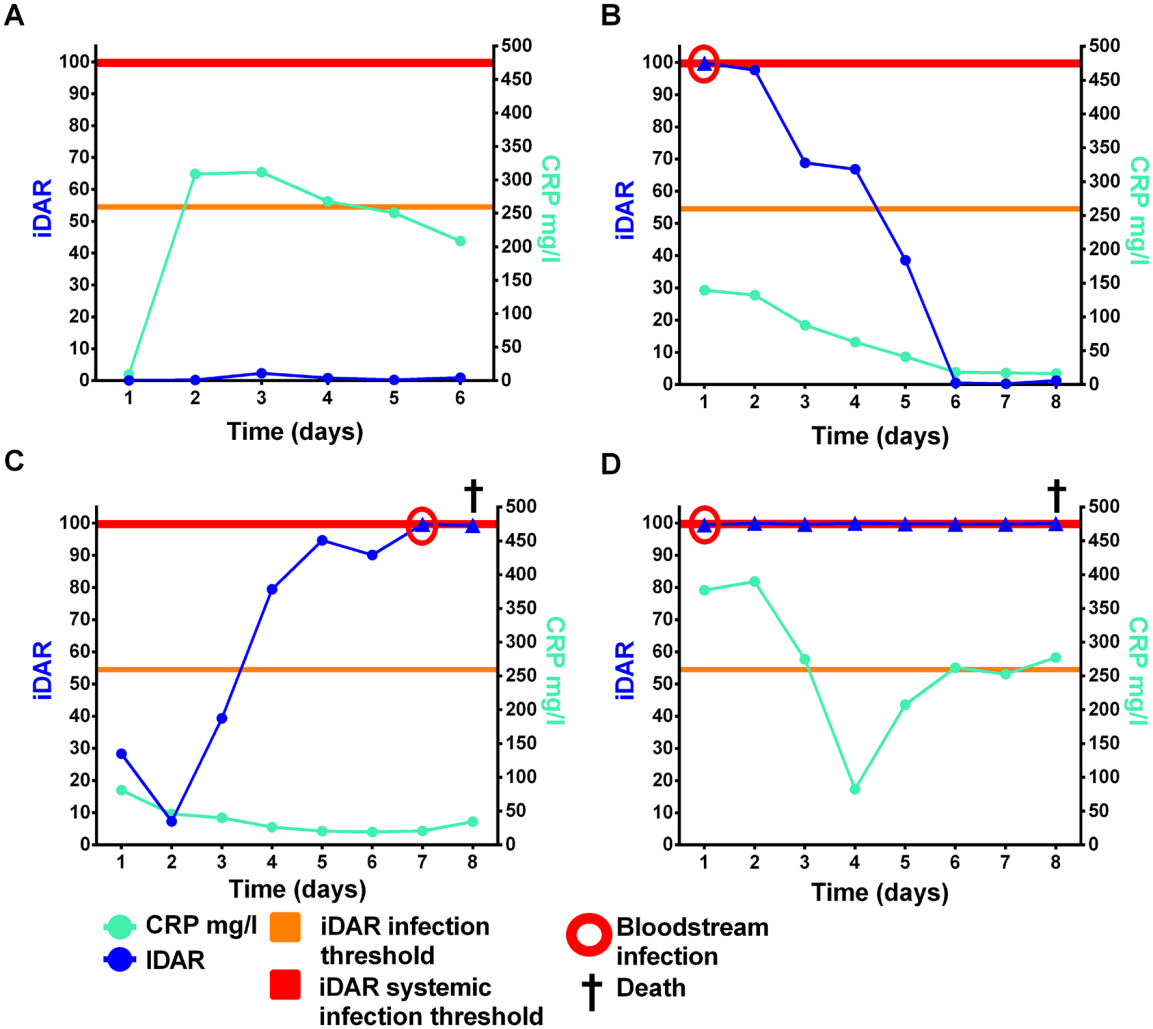
Representative plots of the iDAR score for ICU patients along with CRP comparison. Thresholds of iDAR for infection (orange line) and systemic infection (red line) are indicated by horizontal lines. The timing of positive hemoculture is indicated by a red circle. Patient (**A**): suffered a high velocity crash. Hospital stay was marked by an inflammatory syndrome, which resolved spontaneously without documented infection. iDAR remained low, while CRP levels increased to high concentrations (> 300 mg/l). Patient (**B**): was admitted to ICU for septic shock. Blood culture revealed the presence of *Streptococcus anginosus* immediately treated with moxifloxacin. The clinical course was favorable, with a downward trend of both the iDAR score and CRP levels. Patient (**C**): experienced a hemorrhagic shock. Antibiotics were administered for probable sepsis, and the patient’s condition improved temporarily. After another bleeding episode, the blood culture was positive for *Klebsiella oxytoca*. The iDAR oscillated mostly above the infection threshold, suggesting an infectious state. The score was maximal 2 days before the patient’s death, while the CRP level was not interpretable. Patient (**D**) presented a septic shock of respiratory origin with noradrenaline support. *Streptococcus pneumoniae* septicemia was treated with ceftriaxone/clarithromycin. The iDAR remained at a high level from their admission to their death.

Daily iDAR scoring could be used in intensive care unit patients to ensure swift diagnosis of BSI and to monitor treatment efficacy and clinical course. It has been documented that C-reactive protein (CRP) is commonly used as a marker of acute inflammatory syndrome, and its plasma concentration has always been related to the clinical course of the infection, with decreasing levels suggesting resolution of the infection^38^. For this purpose, the chronological progress of iDAR and CRP results was assessed in ICU patients (see Fig. 6 for detailed monitoring of 4 representative patients).

## Discussion

In recent years, the number of papers describing the application of ML in the medical field has been growing exponentially, particularly in the critical care area. As intensive care unit clinicians are overwhelmed with increasing amounts of data collected at higher speeds, ML is expected to become essential to develop clinical diagnostic and prognostic applications. Computer-based ML tools can be helpful for physicians dealing with complex and challenging cases of bacteremia/sepsis. Several studies have already used risk score models based on EMR data for sepsis detection in the ICU^39,40^*.* To date, clinical decision support (CDS) tools based on machine learning embedded into real-time medical records have been reported^41–43^ and allow fast recognition of the onset of sepsis before overt clinical signs^19,44–50^. In that regard, CDS can reduce the time to antibiotic administration and length of hospital stay and may reduce mortality^28,51^.

This study provides clinical evidence of the prognostic information provided by myeloid immunophenotypic markers in ICU patients using XGBoost analysis. We have developed a scoring algorithm designed to identify systemic infection in patients suspected of bacteremia. The robustness of iDAR can be explained, at least in part, by the large number of predictors from diverse antigens expressed on the surface of blood myeloid cells.

iDAR was able to distinguish systemic infection from localized infection and aseptic inflammation with great precision. The predictive power was excellent (AUC > 0.9) in cross-validation and internal validation of an independent cohort of patients; thus, the score is capable of recognizing a systemic infection well in advance of microbiological culture. The score is calculated from 101 biological measurements and is superior to existing biomarker detection methods. The results confirm that a localized infection can be distinguished from a systemic infection using flow cytometry data obtained from monocytes, dendritic cells and neutrophils. In effect, we achieved an estimated 85% accuracy in the validation phase even though we did not model a localized infection in the discovery cohort. Localized infection can be considered a transition between the noninfectious and bacteremic phenotypes that were tested in our work. Choosing the correct iDAR threshold above which the patient is considered bacteremic will yield a range of sensitivities and specificities. However, it may well be that localized infection is not distinguished from bacteremia by a number of other blood and/or clinical parameters.

Given the impact of timely initiation of antibiotic treatment on outcome, there is a significant interest in predicting BSI at critical time points^52,53^. The iDAR score is a score that can be considered to predict a range of disease probability. The probability of disease can then be converted into clinical monitoring of the patient. To this end, cohorts of healthy individuals and patients harboring cardiac surgery-induced inflammation were compared to systemically infected patients. The inclusion of cardiac surgery in our cohort (2, 24 and 48 h postsurgery) is critical, as it provides comprehensive information on inflammatory kinetics in subjects with no suspicion of infection and thus allows for the recognition of markers, and combinations thereof, which are specific to infection.

We compared iDAR performance with the documented efficacy of procalcitonin (PCT), a common surrogate biomarker of bacterial infection^13^. Using a score of 54.5 as the cutoff, iDAR achieved a sensitivity of 97 and a specificity of 93 compared to PCT sensitivity and specificity of 77% and 79%, respectively. Another widely used and studied biomarker, CRP, is a nonspecific inflammatory biomarker that is also released under noninfectious conditions. CRP is known to be less accurate than PCT in the detection of localized or systemic infection^54^. Indeed, CRP in our study proved to be very ineffective in discriminating infection from inflammation. We also compared the sensitivity and specificity of iDAR with lactate levels > 181 mg/l^55,56^*.* Our findings are consistent with previous results that showed a specificity of 89% and a sensitivity of 25% for lactate to detect systemic infection (data not shown).

The present study confirms and extends previous findings on monocyte and DC response to infection. As described previously^57^, the numbers of intermediate and nonclassical CD16^+^ monocyte subsets increased during BSI as well as in postsurgery aseptic inflammation, together with a global increase in CD16 expression. We were also able to identify a CD91^low^ monocyte subpopulation that was larger under both conditions. A phenotypical and functional characterization of this latter cell subset is ongoing in our laboratory (manuscript in preparation). The β7^+^ fraction of intermediate monocytes was significantly larger in BSI patients than in heart surgery patients and thus seems to expand specifically in response to infection. β7 integrin is implicated in tissue homing, and its upregulation might favor clearance of microorganisms by activated monocytes^58,59^. We also observed a decrease in circulating DCs in both septic and aseptic inflammation, although the reduction was more significant in BSI for the myeloid subset. Markedly reduced numbers of circulating DCs have also been observed in sepsis in previous studies^60–62^. Prolonged persistence of HLA-DR downregulation has been identified as a prognostic factor for mortality^63^ and generally interpreted as an indicator of monocyte anergy and immunoparalysis. In itself, HLA-DR downregulation is not sufficient to discriminate sepsis from other causes of inflammatory states^64^. Indeed, we observed that the loss of HLA-DR expression on monocytes was present in both BSI and postsurgery inflammation. With regard to CD14 expression, most studies have shown that CD14 expression is upregulated in sepsis. However, it was later reported that only soluble CD14 levels are upregulated^65,66^, while membrane CD14 expression is downregulated^67^. In our study, the level of CD14 expression on classical and intermediate monocytes was significantly lower in systemic infection patients than in cardiac surgery patients. In contrast, in nonclassical monocytes, CD14 expression was significantly higher in BSI patients than in cardiac surgery patients.

A large number of studies have identified neutrophil expression of CD64 as a candidate biomarker for bacterial infection and sepsis. However, a wide variation in the performance of CD64 can be observed, depending on the study design. In 2021, Cong et al. compiled 20 studies and found that the pooled sensitivity and specificity were 0.88 (95% CI 0.81–0.92) and 0.88 (95% CI 0.83–0.91), respectively^68^. Our results suggest that CD64 has > 80% specificity, but its sensitivity is modest (70%) in distinguishing infectious from noninfectious patients and even low (59%) in discriminating localized versus systemic infection. When used in combination with other sensitive markers, CD64 may contribute to the clinical diagnosis of sepsis by virtue of its high specificity. CD123-positive neutrophil subsets identified by flow cytometry have proven to correlate with bacteremia/sepsis. It was recently demonstrated that the activation and phagocytic activity of these neutrophils are decreased in immature CD10^-^CD64^+^CD16^low^CD123^+^ cells, potentially making it a marker of sepsis severity^69^.

Recently, Hildebrand et al. identified soluble Delta-like canonical Notch ligand 1 (DLL1) released from monocytes as a predictive marker for sepsis, with a performance superior to that of CRP and PCT^70^. It has also been suggested that Notch signaling is involved in regulating monocyte cell fate^71^. Whether the changes in the monocyte phenotype described here during BSI are associated with increased Notch signaling remains to be further studied.

Several ML algorithms used for predicting BSI have been reported previously and were reviewed by Eliakim-Raz et al. in^72^. These models rely to a large extent on data commonly available in electronic medical records (EMRs). Of note, none of these models is actually implemented in clinical practice, which is partially attributable to their modest predictive power being in the range of 0.6 to 0.83. Ratzinger et al. designed machine learning algorithms for the detection of systemic infection using 21 clinical and laboratory features and achieved an AUROC of 0.73 with a random forest classifier^73^. More recently, Roimi et al. used EMR data to predict bacteremia in ICU patients and developed an ML model with high accuracy (AUC of 0.87–0.93) for internal validation. In the external evaluation process, the performance of their model deteriorated, yielding an AUC of 0.59–0.60^50^. In the present study, we show that specific analysis of innate immune cell phenotype, which is not readily available in routine patient care, provides better accuracy in BSI prediction, however at a higher cost, than basic EMR data.

The SOFA score is a valuable tool for sepsis patient stratification, and quantification of the degree of organ dysfunction^74^. Between the iDAR and SOFA scores, the moderate positive correlation with a coefficient of 0.55 shows a level of significance (*p* < 0.05), which may suggest a linear relationship. Hence, the iDAR score may reflect the progress of organ dysfunction, such that a decrease in the iDAR score would be associated with an improved outcome.

Of note, we did not exclude patients with hematological malignancies, with immune suppression and receiving myeloablative chemotherapy, all factors that would affect myeloid cell phenotype independent of infection or inflammation. Indeed, we aimed to create a model association of BSI regardless of patient characteristics that was closer to real-life practice. It is also interesting to note that the iDAR score achieved a very impressive classification accuracy (ROC = 0.988) with myeloid expression markers without including EMR data. It is likely that additional information, whether clinical or biological, would further refine the prognostic power of the score. However, clinicians should be cautious about using this additional information to support the management of patients with the cutoff values reported in this study. As it is crucial to test the model with larger datasets in future studies, further tailoring of the cutoff values is expected.

There are some limitations in this study that should be addressed. First, the iDAR score has not been validated off-site, although it was subjected to an internal (tenfold cross-validation) and on-site, out-of-sample, validation study. Additionally, the model is derived from a relatively small patient cohort, and thus, our observations should be considered preliminary. However, modern statistical computation power can overcome this problem by performing several bootstraps from the dataset. Repetitive sampling may approximate the true population data, thus saving time and money. Our dataset is somewhat unbalanced due to the small number of cardiac surgery patients at 2 and 48 h, which represent 8.8% and 17% of the dataset, respectively. We investigated the effect of discovery cohort size on the misclassification rate. We observed that the misclassification rate remained constant up to one-third less data, suggesting that the imbalance does not have a significant impact on classifier performance.

Next, regarding the quality of the dataset, some variables have missing values, such as MFIs of nonclassical monocytes and dendritic cell subsets, which tend to decrease sharply in infectious states. For this purpose, MFI values expressed on the cell surface of dendritic cells were not considered.

ICU patients were included on the same day that the blood culture was reported to be positive, and thus, the phenotype does not correspond to the onset of bacteremia but corresponds to the bacteremic state 24 to 48 h after onset, during which 72% of patients received empiric antibiotic therapy. In addition, all heart surgery patients received prophylactic cephalosporin treatment. There is also evidence that many antibiotics directly modulate the immune system in addition to their own antimicrobial properties^75^. Thus, previous antibiotic treatment may influence not only the course of the infection in ICU patients but also the immunophenotypic changes in both ICU and cardiac patients. This potential bias was not taken into account in our study. However, when testing the influence of antibiotic therapy in BSI patients, we found that the determinants of the model were not modified, i.e., prior antibiotic administration did not alter the immunophenotypic shifts of monocytes.

Furthermore, it is likely that iDAR would not be reliable in patients with hematological malignancies or who are receiving immunosuppressive or cytotoxic chemotherapy since various myeloid cell populations would be modified independently of infection response. Although such patients have not been excluded from the dataset, a specific evaluation of the predictive power of the iDAR score in these patient categories should be conducted. Additionally, it is unclear whether the score can be used in patients with low levels of monocytes. In our cohort, the 5^th^ percentile absolute monocyte count was 390 cells/µl.

Finally, a technical requirement for the application of iDAR scoring is the day-to-day reproducibility of MFI measurements using a flow cytometer and the standardization of flow cytometers between different hospital labs. One way to circumvent this problem may be to report the MFI of cell population markers relative to fluorochrome-labeled calibration beads^76^. Alternatively, the Euroflow consortium (www.euroflow.org) has developed a detailed approach for flow cytometer settings that allows for standardized acquisition of 8-color panels across platforms. Through this process, a comparison of MFI-based studies can be carried out at multiple sites. We also acknowledge that our case-finding strategy was limited to cases of BSI and heart surgery in adults; therefore, iDAR has neither been trained nor validated with pediatric subjects.

The analysis of the myeloid cell phenotype described here is quite complex and may not be currently envisioned as point-of-care testing, such as the applications of CRP and PCT. However, progress in automation of flow cytometry procedures allows for the implementation of the iDAR score in routine patient care with a turn-around time of less than one hour, provided that the tests are correctly prioritized in a central laboratory organization. With adequate cutoff values, the iDAR score would quickly identify BSI patients before their condition deteriorates into sepsis.

## Conclusion

Detecting the onset of systemic infection in ICU facilities is an extremely challenging task. The iDAR algorithm is based on the myeloid cell subset phenotype computed by a machine learning approach and may help in assessing the risk of sepsis onset and progression in critical patients.

## Supplementary Information

Supplementary Information.
