# Supplementary material for: Machine learning identification of specific changes in myeloid cell phenotype during bloodstream infections
Source: Sci Rep. 2021 Oct 13;11:20288. doi: 10.1038/s41598-021-99628-8 (PMC8514545; doi:10.1038/s41598-021-99628-8)
Supplement: Supplementary file 1 — Supplementary Information. [file 41598_2021_99628_MOESM1_ESM.docx]

**Machine learning identification of specific changes in myeloid cell phenotype during bloodstream infections**

**Christian Gosset^1, 2*^, Jacques Foguenne^2^, Mickaël Simul^2^, Olivier Tomsin^2^, Hayet Ammar^3^, Nathalie Layios^3^, Paul B. Massion^3^, Pierre Damas^1, 3^, André Gothot^1, 2^**

| **Rank** | **Importance** | **Average Log-Likelihood** | **Predictor** |
| --- | --- | --- | --- |
| 1 | 100.00 | 0.04716 | MFI CD14 CD14^+^β7^-^CD16^+^ |
| 2 | 75.54 | 0.03769 | MFI CD64 GRANULOCYTES NEUTROPHILS |
| 3 | 51.26 | 0.02829 | MFI CD64 CD14^low^β7^-^CD16^+^ |
| 4 | 12.64 | 0.01334 | % MYELOID DENDRITIC CELLS/MONOCYTES |
| 5 | 6.87 | 0.01111 | MFI INTEGRIN β7 CD14^+^CD91^low^ |
| 6 | 3.88 | 0.00995 | CELL COUNT MYELOID DENDRITIC CELLS |
| 7 | 3.11 | 0.00965 | MFI CD14 CD14^low^β7^-^CD16^+^ |
| 8 | 2.24 | 0.00931 | % CD14^+^CD91^low^ |
| 9 | 2.11 | 0.00926 | MFI HLA-DR CD14^+^CD91^low^ |
| 10 | 2.01 | 0.00922 | MFI CD123 GRANULOCYTES NEUTROPHILS |
| 11 | 2.01 | 0.00922 | MFI CD16 MONOCYTES |
| 12 | 1.69 | 0.00910 | CELL COUNT GRANULOCYTES NEUTROPHILS |
| 13 | 1.64 | 0.00908 | MFI CD14 CD14^+^β7^+^CD16^+^ |
| 14 | 1.36 | 0.00897 | MFI HLA-DR CD14^+^β7^+^CD16^+^ |
| 15 | 1.26 | 0.00893 | MFI HLA-DR CD14^+^β7^-^CD16^low^ |
| 16 | 1.24 | 0.00892 | MFI CD91 CD14^+^β7^-^CD16^-^ |
| 17 | 1.17 | 0.00890 | MFI CD16 CD14^+^β7^+^CD16^+^ |
| 18 | 1.11 | 0.00887 | % PLASMACYTOID DENDRITIC CELLS/MONOCYTES |
| 19 | 1.02 | 0.00884 | MFI CD45 MONOCYTES |
| 20 | 1.01 | 0.00883 | MFI CD45 GRANULOCYTES NEUTROPHILS |
| 21 | 0.97 | 0.00882 | MFI CD14 GRANULOCYTES NEUTROPHILS |
| 22 | 0.93 | 0.00880 | CELL COUNT CD14^low^β7^-^CD16^+^ |
| 23 | 0.92 | 0.00880 | MFI CD16 CD14^+^β7^-^CD16^+^ |
| 24 | 0.91 | 0.00880 | MFI INTEGRIN β7 CD14^+^Β7^+^CD16^+^ |
| 25 | 0.89 | 0.00879 | % CD14^+^β7^-^CD16^+^ |
| 26 | 0.87 | 0.00878 | MFI CD16 CD14^low^β7^-^CD16^+^ |
| 27 | 0.85 | 0.00877 | MFI INTEGRIN β7 CD14^low^β7^-^CD16^+^ |
| 28 | 0.85 | 0.00877 | MFI CD123 CD14^+^β7^-^CD16^+^ |
| 29 | 0.78 | 0.00875 | CELL COUNT PLASMACYTOID DENDRITIC CELLS/MONOCYTES |
| 30 | 0.72 | 0.00872 | MFI CD45 CD14^+^β7^+^CD16^-^ |
| 31 | 0.71 | 0.00872 | MFI CD91 CD14^+^β7^-^CD16^low^ |
| 32 | 0.70 | 0.00871 | MFI CD91 CD14^+^β7^-^CD16^low^ |
| 33 | 0.69 | 0.00871 | MFI CD14 MONOCYTES |
| 34 | 0.67 | 0.00870 | MFI HLA-DR GRANULOCYTES NEUTROPHILS |
| 35 | 0.67 | 0.00870 | MFI CD45 CD14^+^β7^-^CD16^-^ |
| 36 | 0.63 | 0.00869 | MFI HLA-DR CD14^+^β7^-^CD16^+^ |
| 37 | 0.62 | 0.00868 | MFI CD14 CD14^+^β7^-^CD16^-^ |
| 38 | 0.60 | 0.00868 | % CD14^+^β7^-^CD16^-^ |
| 39 | 0.60 | 0.00868 | MFI CD45 CD14^low^β7^-^CD16^+^ |
| 40 | 0.58 | 0.00867 | MFI CD123 CD14^low^β7^-^CD16^+^ |
| 41 | 0.57 | 0.00866 | MFI CD64 CD14^+^CD91^low^ |
| 42 | 0.55 | 0.00866 | MFI CD91 GRANULOCYTES NEUTROPHILS |
| 43 | 0.54 | 0.00865 | MFI CD91 CD14^+^CD91^low^ |
| 44 | 0.41 | 0.00860 | MFI CD16 MONONUCLEAR CELLS |
| 45 | 0.40 | 0.00860 | MFI INTEGRIN β7 MONOCYTES |
| 46 | 0.40 | 0.00860 | MFI CD91 MONONUCLEAR CELLS |
| 47 | 0.40 | 0.00860 | MFI CD14 CD14^+^β7^-^CD16^low^ |
| 48 | 0.39 | 0.00860 | MFI CD64 CD14^+^β7^-^CD16^low^ |
| 49 | 0.39 | 0.00860 | MFI CD16 CD14^+^β7^+^CD16^-^ |
| 50 | 0.39 | 0.00859 | MFI CD16 CD14^+^CD91^low^ |
| 51 | 0.37 | 0.00859 | MFI HLA-DR CD14^low^β7^-^CD16^+^ |
| 52 | 0.35 | 0.00858 | MFI CD91 CD14^+^β7^-^CD16^+^ |
| 53 | 0.35 | 0.00858 | MFI CD64 CD14^+^β7^-^CD16^+^ |
| 54 | 0.33 | 0.00857 | MFI INTEGRIN β7 GRANULOCYTES NEUTROPHILS |
| 55 | 0.33 | 0.00857 | CELL COUNT CD14^+^β7^+^CD16^+^ |
| 56 | 0.33 | 0.00857 | % CD14^+^β7^+^CD16^+^ |
| 57 | 0.31 | 0.00857 | MFI INTEGRIN β7 CD14^+^β7^+^CD16^-^ |
| 58 | 0.31 | 0.00856 | MFI HLA-DR MONONUCLEAR CELLS |
| 59 | 0.30 | 0.00856 | MFI CD64 MONONUCLEAR CELLS |
| 60 | 0.30 | 0.00856 | MFI INTEGRIN β7 CD14^+^β7^-^CD16^+^ |
| 61 | 0.30 | 0.00856 | MFI CD16 CD14^+^β7^-^CD16^low^ |
| 62 | 0.29 | 0.00856 | MFI INTEGRIN β7 MONONUCLEAR CELLS |
| 63 | 0.28 | 0.00855 | MFI CD64 MONOCYTES |
| 64 | 0.28 | 0.00855 | MFI CD91 CD14^+^β7^+^CD16^-^ |
| 65 | 0.28 | 0.00855 | MFI CD91 CD14^low^β7^-^CD16^+^ |
| 66 | 0.27 | 0.00855 | % CD14^+^β7^+^CD16^-^ |
| 67 | 0.27 | 0.00855 | MFI CD14 MONONUCLEAR CELLS |
| 68 | 0.26 | 0.00855 | MFI CD16 GRANULOCYTES NEUTROPHILS |
| 69 | 0.25 | 0.00854 | MFI CD64 CD14^+^β7^-^CD16^-^ |
| 70 | 0.25 | 0.00854 | % CD14^low^β7^-^CD16^+^ |
| 71 | 0.24 | 0.00854 | CELL COUNT CD14^+^β7^+^CD16^-^ |
| 72 | 0.23 | 0.00853 | MFI CD64 CD14^+^β7^+^CD16^-^ |
| 73 | 0.23 | 0.00853 | MFI CD14 CD14^+^β7^+^CD16^-^ |
| 74 | 0.21 | 0.00853 | MFI CD45 CD14^+^β7^-^CD16^low^ |
| 75 | 0.21 | 0.00853 | CELL COUNT LYMHOCYTES |
| 76 | 0.20 | 0.00852 | MFI CD14 CD14^+^CD91^low^ |
| 77 | 0.20 | 0.00852 | MFI CD45 CD14^+^β7^-^CD16^+^ |
| 78 | 0.20 | 0.00852 | % CD14^+^β7^-^CD16^low^ |
| 79 | 0.19 | 0.00852 | MFI CD123 CD14^+^β7^-^CD16^-^ |
| 80 | 0.18 | 0.00851 | CELL COUNT CD14^+^β7^-^CD16^-^ |
| 81 | 0.17 | 0.00851 | CELL COUNT CD14^+^CD91^low^ |
| 82 | 0.16 | 0.00850 | MFI CD64 CD14^+^β7^+^CD16^+^ |
| 83 | 0.14 | 0.00850 | MFI HLA-DR CD14^+^β7^+^CD16^-^ |
| 84 | 0.14 | 0.00850 | CELL COUNT MONOCYTES |
| 85 | 0.13 | 0.00849 | MFI CD91 CD14^+^β7^+^CD16^+^ |
| 86 | 0.12 | 0.00849 | MFI CD123 CD14^+^β7^+^CD16^+^ |
| 87 | 0.12 | 0.00849 | MFI CD123 CD14^+^β7^+^CD16^-^ |
| 88 | 0.12 | 0.00849 | MFI CD91 MONOCYTES |
| 89 | 0.11 | 0.00848 | MFI CD16 CD14^+^β7^-^CD16^-^ |
| 90 | 0.10 | 0.00848 | CELL COUNT CD14^+^β7^-^CD16^low^ |
| 91 | 0.10 | 0.00848 | MFI CD45 CD14^+^β7^+^CD16^+^ |
| 92 | 0.10 | 0.00848 | MFI CD45 MONONUCLEAR CELLS |
| 93 | 0.08 | 0.00847 | MFI HLA-DR MONOCYTES |
| 94 | 0.06 | 0.00847 | MFI HLA-DR CD14^+^β7^-^CD16^-^ |
| 95 | 0.06 | 0.00847 | CELL COUNT CD14^+^β7^-^CD16^+^ |
| 96 | 0.05 | 0.00846 | MFI CD123 CD14^+^CD91^low^ |
| 97 | 0.03 | 0.00846 | MFI INTEGRIN β7 CD14^+^β7^-^CD16^low^ |
| 98 | 0.03 | 0.00846 | MFI CD123 MONOCYTES |
| 99 | 0.03 | 0.00845 | MFI CD123 MONONUCLEAR CELLS |
| 100 | 0.02 | 0.00845 | MFI CD123 CD14^+^β7^-^CD16^low^ |
| 101 | 0 | 0.00844 | MFI INTEGRIN β7 CD14^+^β7^-^CD16^-^ |

**Appendix 1.** List of features used within iDAR algorithm.

**
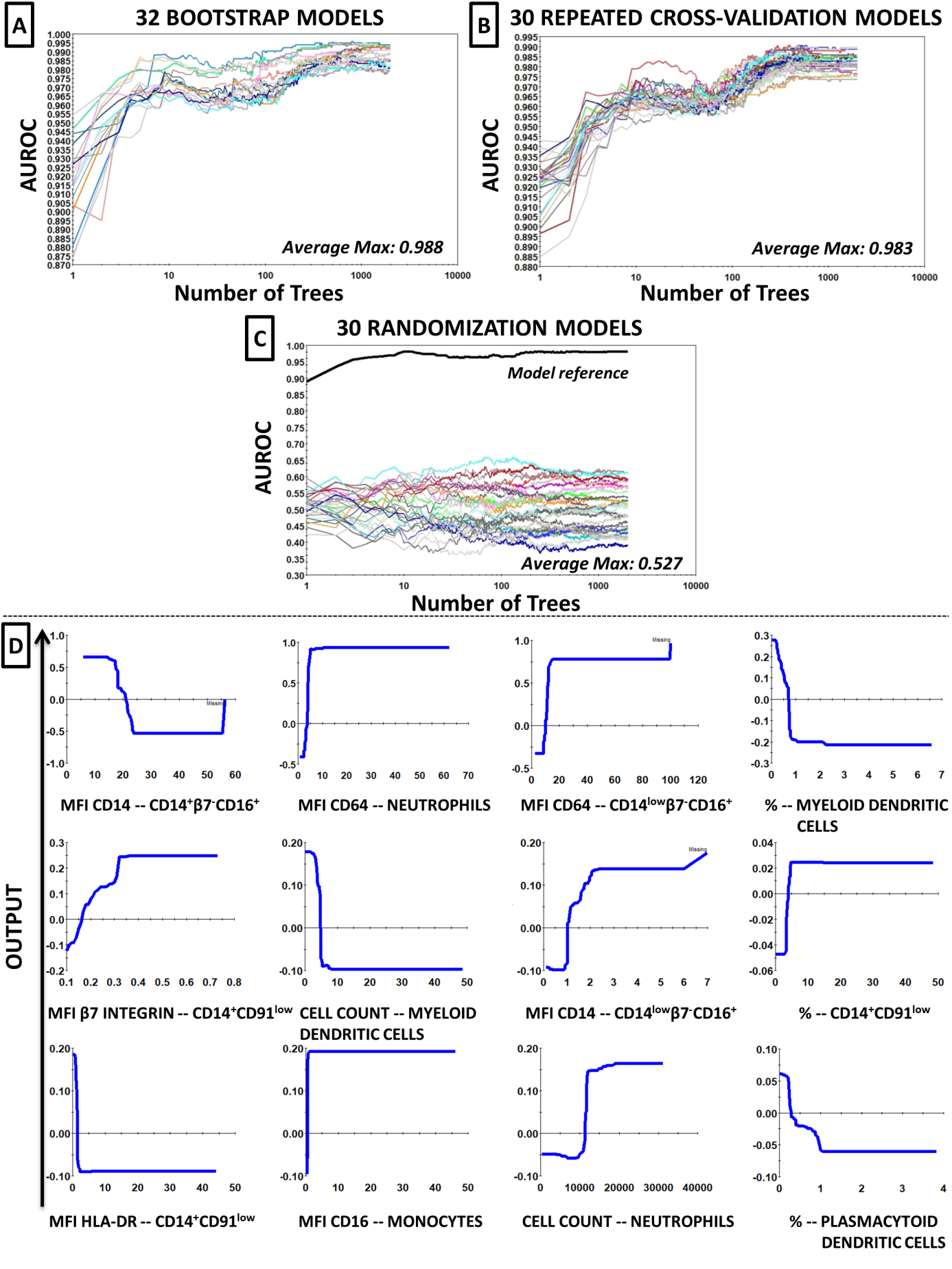
Appendix 2.** Chart curves showing modelling performance for 32 bootstrap (**A**), 30 random seeds repeated cross-validation (**B**) and 30 target shuffle models (**C**). In (**D**), partial dependence plots for the top 12 variables with the variable importance scores as measured by XGBoost.

| **Model Building** | **TOP 10 VARIABLES IMPORTANCE SCORE**  **VIA RANDOMIZATION TEST --- RVIS** |
| --- | --- |
| - **30 REPEATED CROSS- VALIDATION RUNS WITH DIFFERENT SEEDS**   AUROC = 0.98   - **30 RANDOMIZED CLASSIFICATION**   AUROC = 0.58   - **32 PARAMETRIC BOOTSTRAP**   AUROC = 0.988  Specificity = 98  Sensitivity = 92  Threshold = 0.24  **PARAMETERS :**  SUBSAMPLE --- 0.5  LEARNRATE --- 0.001  TERMINAL NODE PER TREE --- 6  PREDICTORS PER TREE --- ALL  PREDICTORS IN MODEL --- ALL  HESSIAN --- 1  LOSS FUNCTION --- CLASSIFICATION BINARY  Newton split search : L₀, L₁, L₂ --- 0  **NO INFECTION**  _(Probability %)_  MEAN --- 0.16  MEDIAN --- 0.07  MIN --- 0.01  MAX --- 3.8  VARIANCE --- 0.17  STANDARD DEVIATION --- 0.41  STANDARD ERROR --- 0.03  **BLOODSTREAM INFECTION**  _(Probability of positive blood culture %)_  MEAN --- 98  MEDIAN --- 99.8  MIN --- 62.9  MAX --- 100  VARIANCE --- 46  STANDARD DEVIATION --- 6.8  STANDARD ERROR --- 0.88 | - MFI CD14 CD14^+^β7^-^CD16^+^   --- 100   - MFI CD64 GRANULOCYTES NEUTROPHILS   --- 69   - MFI CD64 CD14^low^β7^-^CD16^+^   --- 45   - % MYELOID DENDRITIC CELLS/MONOCYTES   --- 12   - MFI INTEGRIN β7 CD14^+^CD91^low^   --- 6   - CELL COUNT MYELOID   DENDRITIC CELLS  --- 3.5   - MFI CD14 CD14^low^β7^-^CD16^+^   --- 2.8   - % CD14^+^CD91^low^   --- 2.4   - MFI HLA-DR CD14^+^CD91^low^   --- 1.9   - MFI CD16 MONOCYTES   --- 1.9 |

**Appendix 3.** The statistics of the randomization classification model, the repeated cross validation runs and the bootstrap are given. The model report includes the parameters used as well as the results of the cross-validation along the first 10 scores of variable importance.

**
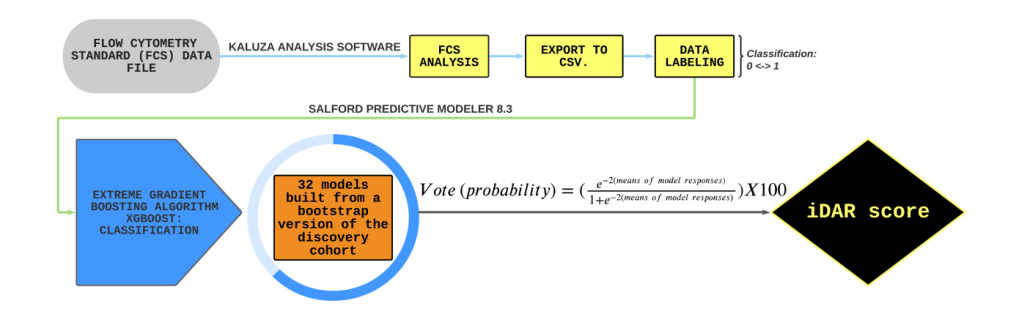
**

**Appendix 4.** iDAR construction flowchart.

**
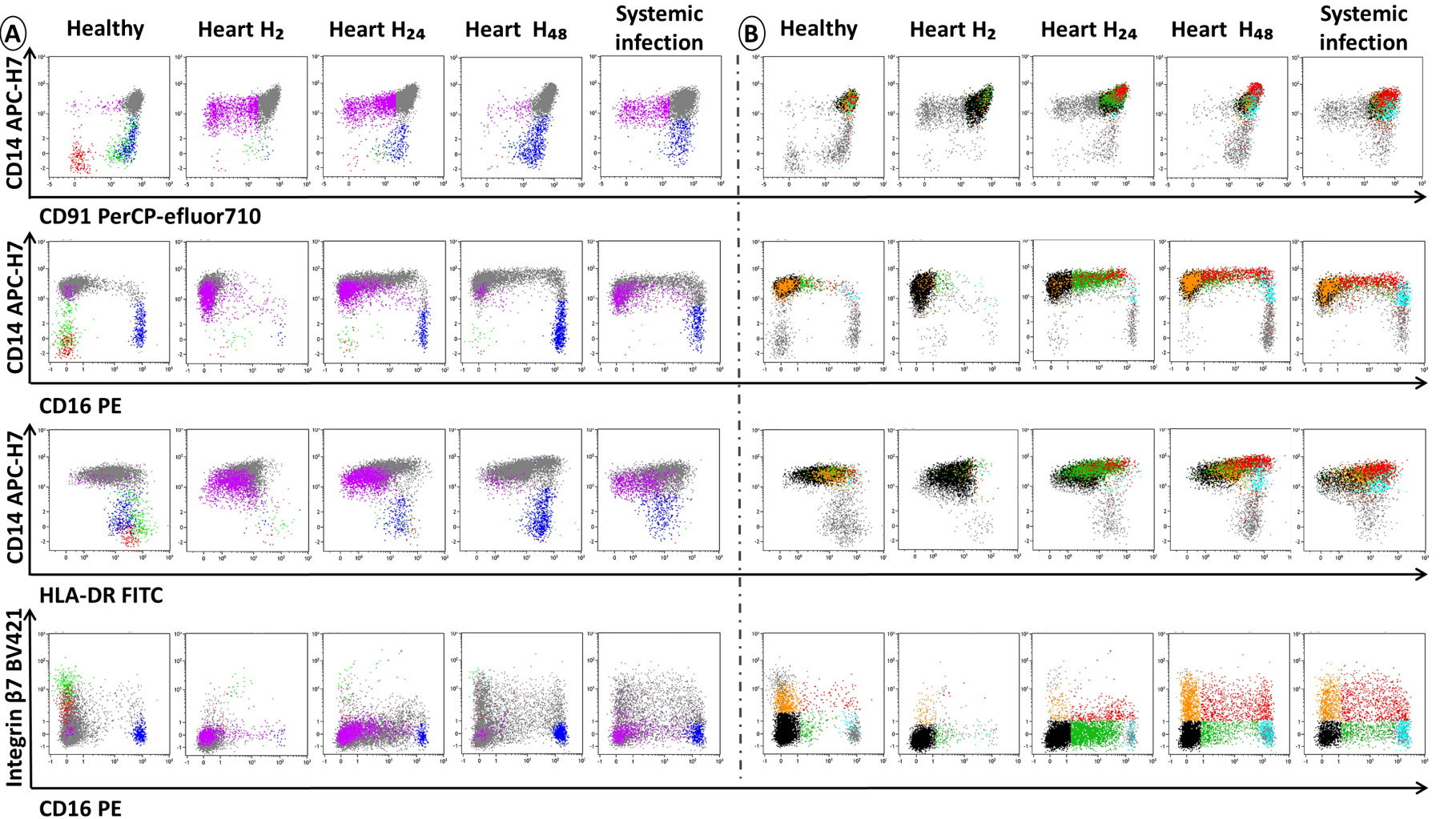
**

**Appendix 5.** Representative flow cytometry bivariate plots of CD14, CD16, CD91, HLA-DR and integrin β7 expression markers are shown for healthy, heart surgery at H₂, H₂₄, H₄₈ and positive blood culture patients. Myeloid populations reported are: plasmacytoid dendritic cells (A; red), myeloid dendritic cells (A; green), CD14^low^ β7^-^CD16^+^ non-classical monocytes (A; blue), CD14^+^CD91^low^ (A; pink), CD14^+^β7^-^CD16^-^ (B; black), CD14^+^β7^-^CD16^low^ (B; green), CD14^+^β7^-^CD16^+^ (B; azure), CD14^+^β7^+^CD16^-^ (B; orange) and CD14^+^β7^+^CD16^+^ cells (B; red).
